# The Effect of Science-Related Populism on Vaccination Attitudes and Decisions

**DOI:** 10.1007/s10865-022-00333-2

**Published:** 2022-06-10

**Authors:** Sarah Kohler, Isabell Koinig

**Affiliations:** 1 Communication and Media Science, Heinrich-Heine-University Düsseldorf, Universitätsstraße 1, 40225 Düsseldorf, Germany; 2 Department of Media and Communications, University of Klagenfurt, Universitätsstr. 65-67, 9020 Klagenfurt, Austria

**Keywords:** Health communication, Vaccination hesitancy, Science-related populism, Vaccination confidence, Collective responsibility

## Abstract

As the COVID-19 pandemic has sadly shown, the decision against vaccination is often linked to political ideologies and populist messages among specific segments of the population: People do not only have concerns about a potential health risk associated with vaccination but seem to have also adopted more populist attitudes towards science. In this study, the relationship between science-related populism and individuals’ attitudes towards vaccination was examined, presuming that scientific-related populism also influences individual responses towards different vaccinations. As different types of diseases and their vaccines might be perceived rather distinctively by the public, different vaccinations were considered. The survey is based on responses from 870 people from Germany and Austria. Results indicate that science-related populism influences responses towards some vaccination types, especially for those that receive extensive media coverage such as COVID-19 and measles (MMR). There was no significant impact of science-related populism on individuals’ vaccination intentions for other vaccines like seasonal influenza, human papillomavirus, or tick-borne encephalitis. In conclusion, limitations and directions for future research are addressed.

**Supplementary Information:**

The online version contains supplementary material available at 10.1007/s10865-022-00333-2.

## Introduction

As the COVID-19 pandemic has sadly shown, the decision against vaccination is often linked to political ideologies and populist messages (Novak, 2021), which are spread even by national governments and political parties (Mede & Schäfer, 2020). Kennedy (2019, p. 513) states, that “a profound distrust in elites and experts” among marginalized segments of the population, is the common denominator of political populism and skepticism towards scientific findings which results in vaccine hesitancy (Edwards et al. 2021; Guay et al. 2019). People who distrust political institutions also distrust science and research and are even prone to conspiracy beliefs (Eberl et al. 2021, p. 280; Stecula & Pickup, 2021). The levels of vaccine refusal have become “alarming” (Roccato & Russo, 2021). In this case, people are not only worried about the possible health risks vaccination might bear, but have also become more skeptical of science and scientific evidence in general (Larson et al. 2011; Badur et al. 2020). This argumentation serves as the basis for anti-vaccine movements to gain more ground (Dubé et al. 2015, 2020).

Scholarly commentaries and journalistic reporting suggest that populist attitude toward science has increased (e.g.; Lehming, 2019; Kastilan, 2021; Deutschlandfunk Nova, 2021), although first studies suggest otherwise (Mede & Schäfer, 2021). Two large surveys in Germany and Switzerland found that trust in science has increased during the COVID-19 pandemic (Wissenschaft im Dialog, 2020; Science Barometer Switzerland, 2020). However, the news coverage on e.g., COVID-19 has experienced a visible growth (Leidecker-Sandmann et al. 2021) and was often connected with individuals’ vaccine hesitancy (The Coconel Group, 2021) and the rise of anti-vaccine-movements in several countries (Speed & Mannion, 2020). In contrast, news coverage on other vaccines often focused more on general information on the vaccine or the transmission of the disease (Calloway et al. 2006). In a content analysis by Casciotti et al. (2014), the authors found hints that the amount of conflicts within news coverage associated with HPV increased and warned, that this might backfire against public health efforts. Faasse et al. (2017) reported, that adverse events reporting was often not related to vaccines but to news coverage, which might increase public concerns about potentially unpleasant or harmful outcomes of vaccinations. Wu et al. (2018) emphasized, that even negative imagery of vaccination in news coverage (e.g., a screaming child receiving an injection, that does not correspond to each vaccination in reality) might lead to increased fears and worries in respondents.

Vaccine hesitancy challenges national healthcare systems throughout the world. As vaccination rates are insufficient, vaccine-preventable diseases have started to reemerge in both developed and developing countries (Habersaat & Jackson, 2020) and can only be put to an end by a sufficiently high vaccination rate. The COVID-19 pandemic has also revealed differences between the individual countries: The Financial Times stated in a news article that vaccination rates in German-speaking countries (including Austria, Germany, and Switzerland) are considerably lower than in the rest of Europe while each of the German-speaking countries also faces an increase in anti-vaccine movements (Jones & Chazan, 2021). Consequently, vaccination hesitancy is likely to have a negative impact on national healthcare systems, leading to increased healthcare costs.

Even though vaccination hesitancy might have become more visible in news coverage opposing measles or COVID-19 vaccination, it emerged as soon as the first vaccination was introduced (The College of Physicians of Philadelphia, 2018). The most prominent historic example concerns the public’s opposition to the smallpox vaccination in the late 1800s when the first anti-vaccination leagues were formed. Later on, the safety of a variety of vaccination was questioned, including diphtheria, tetanus, and pertussis (DTP), and measles, mumps, and rubella (MMR); some of these leagues still exist today (The College of Physicians of Philadelphia, 2018).

In this context, social media occupies a central role in how opinions about vaccination are formed (Koinig & Kohler, 2023). In recent years, social media outlets have been increasingly criticized for facilitating the dissemination of misinformation by anti-vaccine movements. Misinformation thereby alludes to any kind of information that contradicts or even challenges scientific evidence or expert opinions, inducing people to make often fatal health decisions. The Internet in general has been criticized for the increase in misinformation, which can prevent individuals from getting vaccinated altogether (Dubé et al. 2015).

The role of social media is also addressed in research on populism. Engesser et al. (2017) pointed out, that the direct link between social media and the people allows populists to spread their ideologies easily. Facebook and Twitter are understood by populists as the people’s voice which is also accompanied by distrust in mainstream news media (Gerbaudo, 2018; Mavragani & Ochoa, 2018). The distrust is not limited to media only, but also to scientific expertise and evidence and has led to the emergence of a significant number of conspiracy theories (Eberl et al. 2021; Speed & Mannion, 2020; Stecula & Pickup, 2021). These “anti-science sentiments” (Krämer & Klingler, 2020, p. 256) can be described as science-related populism (Mede & Schäfer, 2020). In this context, the populist’s perception of an ‘*academic elite’* as the antagonist to the ‘*ordinary people’* becomes crucial. In their view, the knowledge of the ‘ordinary people’ is superior to scientific methods and evidence as it rests on common sense, everyday experience, and even gut feeling (Mede & Schäfer, 2020; Saurette & Gunster, 2011). As such, the ‘truth’ which is produced by the ‘academic elite’ is perceived as fundamentally detached from the everyday life of the ‘ordinary people’ as scientists seem to be “incapable of providing simple, hands-on solutions that ordinary people demand” (Mede & Schäfer, 2020, p. 481).

Because of this relationship, there are competing claims between ‘the academic elite’ and ‘the ordinary people’ in terms of a *decision-making sovereignty* and a *truth-speaking sovereignty* (Mede & Schäfer, 2020). From the perspective of populists, the ‘academic elite’ claims both forms of sovereignty illegitimately, because its decisions within the process of scientific knowledge production are allegedly biased by third interests (decision-making sovereignty) and because scientific knowledge ignores or even neglects everyday experiences, instead of resting on ‘seemingly alienated theories developed in the proverbial ivory tower’ (Mede & Schäfer, 2020, p. 483, see also Saurette & Gunster 2011).

As we pointed out earlier, science-related populism is rooted in populism and skepticism towards science, scientific actors, and scientific findings. Hence, we presume that it will also influence individual responses towards different vaccinations.

## Science-related populism and antecedents of vaccination

Betsch et al. (2018) developed the 5 C psychological antecedents of vaccination scale which covers a broad variety of attitudes: It concerns individual’s confidence, complacency, constraints, collective responsibility, and calculation (Betsch et al. 2018) when it comes to vaccination. We assume that science-related populism is correlated with these sub-dimensions. To give an example: If someone refuses to get vaccinated against COVID-19 because he/she doubts science and the effectiveness of vaccines in general, this will negatively affect his/her vaccination confidence.

*Vaccination confidence* is a key determinant of individuals’ vaccination intention and includes “trust in (i) the effectiveness and safety of vaccines; (ii) the system that delivers them, including the reliability and competence of the health services and health professionals, and (iii) the motivations of policy-makers who decide on the need of vaccines” (MacDonald 2015, p. 4162). If confidence is high, individuals regard vaccination positively (Askelson et al. 2010). Individuals who exhibit more populist attitudes might regard getting vaccinated as unnecessary or even harmful, and might therefore not believe in the effectiveness of vaccinations at all (Larson et al. 2014; Kennedy, 2019; Roccato & Russo, 2021). This was also shown in a survey among the Australian population. Edwards et al. (2021, p. 4) questioned, whether participants would get vaccinated if there is a “safe and effective” vaccine. They found, that people with populist views were less likely get vaccinated (p. 6). In consequence, we assume:

*H1: Individuals who exhibit higher degrees of science-related populism will have lower vaccination confidence.*

*Complacency* describes the general tendency of people to consider themselves to be less susceptible to (health) risks than others. In connection with vaccination, this means that the dangers of vaccine-preventable diseases are underestimated and one’s own knowledge regarding the effectiveness of vaccines is overestimated (Betsch et al. 2017). The COVID-19 pandemic showed, that people from the anti-vaccine movements even doubt the existence of the virus (Jaspal & Nerlich, 2022). Therefore, we assume, that people with science-related populist attitudes will exhibit high level of complacency, too. Consequently, we propose the following hypothesis:

*H2: Individuals who exhibit higher degrees of science-related populism will have high degrees of complacency.*

*Constraints* can be both structural and psychological in nature, and concern, for instance, access to vaccination, the lack of self-control, or long distances to obtain a vaccination. Constraints are what prevent individuals from getting vaccinated (Betsch et al. 2018). As constraints can be psychological in nature, we assume, that people who are more prone to science-related populist messages, will perceive more constraints. To give an example, Kowalski et al. (2022) examined how people can be motivated to get a booster vaccination against COVID-19 and found, that marginalized groups perceive more constraints. This leads us to hypothesize:

*H3: Individuals who exhibit higher degrees of science-related populism will perceive more constraints to vaccination.*

*Collective responsibility* presumes that individuals see the “broader picture”, i.e. they assume that through collective action, a potential health problem can be solved (Betsch et al. 2018). This suggests, that individuals might decide to get vaccinated because they see the benefit for their peers and community, rather than their own benefit (Betsch et al. 2017). Research has shown, that e.g., right-wing voting intentions are associated with incivility (Frischlich et al. 2021) and anti-social personality traits (Enders & Uscinski, 2021). We assume, that such predispositions are also prevalent in science-related populism. If collective responsibility is low, individuals will feel less inclined to get vaccinated. Therefore, we propose the following hypothesis:

*H4: Individuals who exhibit higher degrees of science-related populism will have low levels of collective responsibility.*

*Calculation* describes the time and energy individuals invest in searching for relevant information on the vaccine. The information is then used as a basis for decision-making: highly calculated individuals have been found to oppose vaccination, while less calculated individuals are expected to favor vaccination (Betsch et al. 2018). In this case, we assume that calculation will not be affected by science-related populism.

## Method

In order to test our hypotheses, we conducted an online survey with participants from Germany and Austria (n = 870). We collected our data via Clickworker during the ongoing COVID-19 pandemic in May and June 2020.

### Measures

We used the *SciPop Scale* (KMO = 0.834, p = .000) by Mede et al. (2020) which measures populism towards science on four dimensions: conceptions of the ordinary people (“what unites the ordinary people is that they trust their common sense in everyday life”; “ordinary people are of good and honest character”), conceptions of the academic elite (“scientists are only after their own advantage”, “scientists are in cahoots with politics and business”), demands for decision making sovereignty (“The people should have influence on the work of scientists”; “People like me should be involved in decisions about the topics scientists research”), and demands for truth-speaking sovereignty (“In case of doubt, one should rather trust the life experience of ordinary people than the estimations of scientists”; “We should rely more on common sense and less on scientific studies”). Participants were asked to answer whether they agreed with the statements on 7-point Likert scales ranging from (1) ‘I completely disagree’ to (7) ‘I fully agree’.

Betsch et al. (2018) developed the *5 C Psychological Antecedents of Vaccination Scale*, which measures antecedents of vaccination with five subdimensions. We used the long version with three items for each subdimension: Confidence (e.g., “I am completely confident that vaccines are safe”; KMO = 0.732, p = .000), complacency (e.g., “Vaccination is unnecessary because vaccine-preventable diseases are not common anymore”; KMO = 0.682, p = .000), constraints (e.g., “Everyday stress prevents me from getting vaccinated”; KMO = 0.711, p = .000), calculation (e.g., “When I think about getting vaccinated, I weigh benefits and risks to make the best decision possible”; KMO = 0.702, p = .000), and collective responsibility (e.g., “When everyone is vaccinated, I don’t have to get vaccinated, too”; KMO = 0.650, p = .000). Participants were asked to answer three questions for each subdimension whether they completely disagree (1) or fully agree (7) with the statement.

#### Additional measures

In order to not generate isolated findings that only apply to the COVID-19 vaccine, we reported *information for several vaccinations*. We assume that different types of diseases and their vaccines are perceived rather distinctively by the public, e.g., diseases which are transmitted by humans might have a higher impact on collective responsibility than diseases which are transferred by animals only. Further, if there were visible public debates on vaccination hesitancy for a specific vaccine like COVID-19 or MMR (measles, mumps, rubella), this might lead people to question the vaccination and its effectiveness more frequently than those vaccinations which were less or only seasonally discussed in the news. Therefore, we selected two vaccinations, which were excessively discussed in news coverage^1^ (MMR; COVID-19), one vaccination which is transmitted by animals (tick-borne encephalitis; TBE), one vaccination which is almost non-visible in news coverage (meningococcal disease; MD), one, which is only periodically discussed (seasonal influenza; SI), and one, which is known to have a quite low vaccination status (human papillomavirus; HPV).

We measured *Health Consciousness* (Gould, 1990) with six items on a 7-point scale from (1) ‘I completely disagree’ to (7) ‘I fully agree’ (e.g.; “My health depends on how well I take care of myself.”) as well as *Health Self-Efficacy*. The latter was established via 4 items and was based on Rimal (2001) (e.g., “I consider myself capable of taking care of my body.”).

*Health Information Seeking Behavio*r (Weaver et al. 2010, e.g., “I like to gather as much information on health as I can before making a decision”) and *Health Information Orientation* (DuBenske et al. 2009; e.g., “The amount of health information available today makes it easier for me to take care of my health”) were also measured using a 7-point Likert scale ranging from completely disagree (1) to fully agree (7).

### Participants

Respondents were invited via the online recruitment platform Clickworker in May and June 2021 and received € 1.00 for finishing the short questionnaire (approximately 7 min). We recruited participants from Austria and Germany and asked individuals also for their nationality. The total sample consisted of 532 Austrians and 300 Germans, 38 people stated other nationalities. Participants were between 18 and 69 years old (M = 37.43; SD = 10.82). The gender distribution was almost equal (51% female), one third of respondents indicated having children (30.1%). In terms of education, the sample was biased toward people with higher education (high school degree: 28%; university degree: 40%) (Table 1).

**Table 1:** Participant characteristics (n = 870)

		N	%
Gender	female	445	51.1
	male	420	48.2
	diverse; other	6	0.7
Age, yrs., mean (SD)		37.4	(10.8)
Education	professional school	223	25.7
	high school degree	224	25.7
	university degree	382	43.9
Children	yes	262	30.1

The vaccination status differed depending on the vaccination type inquired (see Table 2). The largest part of the participants was vaccinated against measles, mumps, and rubella (MMR; 78.3%). Around one-third had already received at least one vaccination against COVID-19 (34.2%), tick-borne encephalitis (TBE; 29.3%), and meningococcal disease (MD; 30.4%). Only 22.4% was vaccinated against seasonal influenza (SI) and only 13.2% against human papillomavirus (HPV). With regard to COVID-19, the vaccination rate reflected the status quo of June 2021, since the national vaccination campaigns and vaccination itself were still ongoing. Further, we did not ask when the last seasonal influenza shot was received, therefore, participants might have received a dose at any time during the past years. It is noteworthy that a significant amount of people was unsure as to whether they were vaccinated against HPV (26.6%) or MD (24.5%)

**Table 2:** Vaccination status of participants (n = 870) in %; June 2021

	yes or initiated (1 dose min.)	unsure
COVID-19	34.2	1.5
HPV	13.2	26.6
MMR	78.3	7.3
TBE	29.3	17.7
MD	30.4	24.5
SI	22.4	2.8

### Procedure

After granting informed consent, participants were asked to provide answers to some general information on the topic of the survey (e.g., individual health behaviors and attitudes towards science). Additional questions determined individuals’ health consciousness and health information-seeking behaviors. Afterward, we asked which information sources had the highest relevance when looking for health information (e.g., friends, family, social media). Then, participants answered questions about their attitudes towards science and vaccination. We also inquired whether they were vaccinated against several diseases.

Finally, we asked some basic demographic questions regarding participants’ age, gender, education, and whether they had children.

We pre-tested the survey instrument with a convenience sample of 25 students in a university course on quantitative methods in social sciences for the comprehensibility and functionality of the online survey.

### Data Analysis

We coded the vaccination status for each vaccination as a dichotomous variable (vaccinated yes/no) and did not include those subjects, who were unsure about their vaccination status or refused to answer the question. We aggregated science-related populism (SciPop) and each subdimension of the 5 C using mean values. Then, we performed binomial logistic regressions with the vaccination status for each vaccine as the outcome and SciPop and control variables (health information seeking, health consciousness) as predictors. For the logistic regression, we tested linearity using the Box-Tidwell procedure (Box & Tidwell, 1962). Moreover, we applied Bonferroni-correction to all interaction terms in the model (Tabachnick & Fidell, 2018). All variables were found to follow a linear relationship. Further, multicollinearity was not a confounding factor in the analysis, since the correlations between the predictor variables were low (r < .60) (according to Pituch & Stevens 2019, p. 77).

Afterward, we conducted regression analyses to test for the impact of science-related populism and control variables on the antecedents of vaccination. We report two-sided *p* values for all tests. All analyses were conducted using SPSS version 26.

## Results

### Science-related populism and vaccination status

We analyzed whether the vaccination status of each vaccination correlated with respondents’ science-related populism. Hence, we performed a logistic regression analysis for each vaccination (Table 3).^2^ Of the six variables entered into the regression models, SciPop contributed significantly to reducing the likelihood of getting vaccinated against COVID-19 (OR = 0.773) or MMR (OR = 0.602). There was no significant impact of SciPop on individuals’ vaccination intentions for the other vaccines (TBE, HPV, SI, MD). Although age seemed to be significantly related to MMR, TBE, and HPV, the Odds Ratio was always close to 1. This means, that there is no association in predicting the likelihood of getting vaccinated. Health Consciousness was found to have a positive effect (OR = 1.596), increasing the likelihood of getting vaccinated against MMR, and so did Health Information Seeking Behaviour (OR = 1.355) for HPV. All the other variables had no significant effect.

**Table 3:** Binomial logistic regression of vaccination status and science-related populism

		Covid-19	MMR	TBE	HPV	SI	MD
age	OR	1.013	0.947***	0.940***	0.884***	1.005	0.91
	CI	[1.00, 1.02]	[0.92, 0.96]	[0.92, 0.95]	[0.85, 0.91]	[0.99, 1.02]	[0.89, 0.92]
gender	OR	0.899	1.484	1.057	1.273	0.881	0.886
(1 = female)	CI	[0.67, 1.20]	[0.96, 2.27]	[0.76, 1.46]	[0.79, 2.03]	[0.63, 1.22]	[0.62, 1.26]
education	OR	1.08	0.901	1.043	1.185	1.034	0.939
	CI	[0.92, 1.26]	[0.71, 1.13]	[0.87, 1.24]	[0.92, 1.51]	[0.86, 1.23]	[0.77, 1.13]
SciPop	OR	0.773***	0.602***	0.884	0.988	0.907	0.833
	CI	[0.67, 0.88]	[0.49, 0.72]	[0.76, 1.02]	[0.81, 1.20]	[0.78, 1.05]	[0.71, 0.97]
Health	OR	1.061	1.596***	1.15	0.788	1.146	1.14
Consciousness	CI	[0.86, 1.30]	[1.16, 2.19]	[0.90, 1.46]	[0.57, 1.08]	[0.90, 1.45]	[0.88, 1.46]
Health	OR	0.959	0.948	1.126	1.355*	1.032	0.908
Information Seeking	CI	[0.80, 1.14]	[0.72, 1.24]	[0.91, 1.38]	[1.02, 1.80]	[0.84, 1.26]	[0.72, 1.13]
Nagelkerke’s R²		0.036	0.144	0.120	0.246	0.009	0.234

OR = Odds Ratio; CI = Confidence Interval (95%); Odds Ratio between exposure and outcome (OR > 1 greater odds; OR = 1 no association; OR < 1 lower odds); Dependent variable: Vaccination status (1 = vaccinated, 0 = not vaccinated); *p < .05 **p < .01 ***p < .001

### Effect of science-related populism on vaccination attitudes

In a second step, we aimed to investigate the relationship between science-related populism and the 5 C antecedents of vaccination (confidence, complacency, constraints, calculation, collective responsibility). We proposed four hypotheses, in which higher degrees of science-related populism are negatively associated with individuals’ vaccination confidence (H1) and collective responsibility (H4), but positively associated with individuals’ levels of compliance (H2) and perceived constraints (H3). We assume, that there is no association between calculation and science-related populism.

We ran linear regression models for each subdimension and included all participants. We initially considered to only focus on those individuals, who reported to be not vaccinated at least for one disease. Yet, it turned out, that only 15 people were vaccinated against all diseases. This means, that the vast majority has no complete vaccination record. This probably results from the fact, that only one-third of the participants was already vaccinated against COVID-19 at the time the study was conducted (May/June 2021).

**Table 4:** Linear regression analyses (n = 721)

	Confidence	Complacency	Constraints	Collective Responsibility	Calculation
F-Test	F(7) = 30.89; p < .001	F(7) = 60.61; p < .001	F(7) = 17,13; p < .001	F(7) = 38,67; p < .001	F(7) = 16,71; p < .001
	**β**	**β**	**β**	**β**	**β**
age	− 0.136***	− 0.006	− 0.061	− 0.12***	0.192***
gender (1 = female)	− 0.067*	− 0.059*	− 0.126***	0.075*	0.022
education	0.049	0.034	0.064	− 0.001	0.012
children (1 = yes)	− 0.037	− 0.073*	− 0.051	0.031	− 0.068
SciPop	− 0.420***	0.598***	0.286***	− 0.491***	0.089**
Health Consciousness	0.172***	− 0.011	− 0.148***	0.17***	0.153
Health Information Seeking	0.072	− 0.079*	− 0.057	0.03	0.187
**adj. R²**	**0.225**	**0.367**	**0.136**	**0.268**	**0.133**

All five regression analyses were statistically significant (Table 4). The adjusted R² for the first model with confidence as outcome was 0.225, indicative of a medium goodness-of-fit according to Cohen (1988). SciPop (-0.42) was the strongest predictor, next to Health Consciousness (0.172) and age (-0.136). Therefore, we can confirm hypothesis H1. The higher the level of science-related populism, the lower the level of confidence. The regression model for complacency had a high adjusted R² (0.367). Again, science-related populism turned out to be the strongest predictor (0.598), while gender (-0.059) and Health Information Seeking (-0.079) were significant, but with very low standardized beta-coefficients. We can confirm hypothesis H2, as the higher the level of science-related populism, the higher the level of complacency. Hypothesis H3 proposed a relationship between constraints and SciPop. Still, SciPop was the strongest predictor (0.286) next to gender (-0.126) and Health Consciousness (-0.148). We can also confirm hypothesis H3. The analysis of collective responsibility showed an explained variance of 26.8% (R² = 0.268). The coefficients of age (-0.12) and Health Consciousness (0.17) were significant but very low. Science-related populism was the strongest predictor in this regression (-0.491). Therefore, we can confirm hypothesis H4, which postulated that the higher the level of SciPop, the lower the level of collective responsibility. The last regression model used calculation as outcome, for which we did not propose a hypothesis. In this case, science-related populism was significant, but with a low coefficient (0.089). Age (0.192) turned out to be the strongest predictor in this model. So, our assumption was met: Science-related populism does not strongly affect calculation.

## Discussion

This study examined the relationship between science-related populism and the effects on vaccination attitudes and decisions. Thereby, the study relied on survey data from Germany and Austria which was collected during the COVID-19 pandemic in May and June 2021. We conducted logistic regressions using the vaccination status (yes/no) as outcome and science-related populism, health information-seeking behavior, health consciousness, and demographic variables (age, gender, level of education) as predictors. The individual regressions showed, that there was a significant relationship between science-related populism when it comes to vaccination against COVID-19 or MMR. In both cases, a higher score of science-related populism decreased the likelihood of getting vaccinated. There were no associations between science-related populism and the other vaccines (TBE, SI, MD, HPV).

Our study results imply that vaccination against COVID-19 and MMR is a more polarized issue than vaccinations against other diseases in Germany and Austria. As we initially pointed out, we wanted to compare different vaccinations. As the others were less (TBE) or almost non-visible (MD) in news coverage when compared to COVID-19 or MMR, we assume, that the role of the media is crucial. We suggest, that particularly vaccines, which are debated in public and the media, are subject to science-related populism. This finding is in line with results from other European countries, including Italy (Speed & Mannion, 2020) and Poland (Zuk & Zuk, 2020). As the vaccination against COVID-19 and MMR (to be specific: measles) is at present heavily debated publicly, individuals’ attitudes towards both vaccination types are correlated with science-related populism. In other words: The decision to get vaccinated seems to be connected with publicly discussed vaccination (MMR; COVID-19) but not with vaccinations which are given less space in public and the media. Further, there are no significant differences between vaccinations which have a low vaccination status (HPV) or diseases that are only transmitted by animals (TBE).

In a second step, we conducted regression analyses with science-related populism as predictor and each sub-dimension of the 5 C antecedents of vaccination scale as outcome. Again, science-related populism turned out to be a strong predictor, negatively affecting vaccination confidence and collective responsibility. These two constructs are extremely relevant, given that high confidence leads to positive attitudes towards vaccination, while collective responsibility assumes that individuals are inclined to get vaccinated if they see not only their own (individual) but also others’ (group) benefit. If the level of science-related populism is higher, the collective responsibility and confidence in vaccination decreases. At the same time, science-related populism positively affects complacency and constraints. This suggests that individuals who are more drawn to science-related populism might feel that they are less susceptible to the health risks and, therefore, see no need to get vaccinated (complacency), might perceive that obtaining a vaccination is linked to both structural and psychological barriers, such as time and availability (constraints), and might not see the benefits and relevance of vaccinations for the society (collective responsibility).

## Limitations

While the study was innovative, several limitations need to be addressed. The study was conducted via the online recruitment platform Clickworker during the ongoing COVID-19 pandemic and was limited to respondents from Germany and Austria. Therefore, the results are specific for this particular time and the selected countries. For example, the vaccination status for COVID-19 was quite low in June 2021 (35%), but has increased since then to 75% in Austria and Germany (April 2022: ORF, 2022; Bundesministerium für Gesundheit, 2022). The convenience sample upon which our analysis is based is also biased in terms of age, education, and nationality and is neither representative of the Austrian or German population. For future studies, we would suggest striving for a representative sample of the Austrian and German populations. An international comparison with other countries with higher vaccination rates might also be interesting.

During the study, we did not encourage respondents to have a look at their vaccination records. This might explain why a significant number of the respondents did not recall whether they were vaccinated against e.g., MD or not. A more thorough record might be necessary for future studies.

## Conclusions

In our study, we took individuals’ science-related populism and individuals’ attitudes towards vaccination as the basis for our analysis. Science-related populism, as derived from the SciPop Scale (Mede et al. 2020), postulates that conditioned by a far-reaching scientific skepticism, individuals of specific segments of the population have adopted more populist attitudes towards science. Results indicate this assumption to hold true, particularly for COVID-19 and MMR vaccinations in Austria and Germany. In this case, it seems plausible to argue that the sheer amount of media coverage a vaccination receives might ignite and further increase public concern about the respective vaccination. This problem is similar to previous findings which determined that the communication of controversial topics can increase the risk perception and public concern. In this case, we raise the question as to which media coverage might lead to negative attitudes towards vaccination. Negative feelings might be even triggered by vaccination campaigns; therefore, they should be designed with care (Koinig, 2021).

Moreover, the results might also support the assumption that anti-vaccination movements and populist right-wing movements are somewhat related (Zuk & Zuk, 2020). This is the case given that political actors participate in public debates about health issues, which can lead the issue to be associated with polarized responses (Merkley & Stecula, 2020) – which was also sadly shown during the COVID-19 pandemic. This is also in line with previous findings. Fowler & Gollust (2015; also see Gollust et al. 2010; Roccato & Russo, 2021) stated, that controversies in news coverage and the politicization of vaccines results in decreasing support for vaccine requirements and programs as well as decreasing trust in government and doctors.

Likewise, health information on the internet continues to be a problem, given a lack of fact-checking. On the one hand, the ongoing spread of misinformation on the internet by anti-vaccine movements poses a challenge and might negatively impact health policy (Zuk & Zuk, 2020; Speed & Mannion, 2020). Yet, previous research has also found that social media is a viable outlet for both vaccine supporters and vaccine opponents (Milani et al. 2020). Only if information on vaccinations is made available to the wider public, individual’s willingness to get vaccinated can be increased (Nyhan & Reifler, 2010). Yet, people with populist attitudes often distrust mainstream media which is also expressed by the slogan “liar press (‘Lügenpresse’)” (Haller & Holt, 2019, p.1667). Roccato & Russo (2021, p. 2) explain, that populists and COVID-19 vaccine refusers prefer social media as a source of information. Therefore, if vaccination campaigns try to convince anti-vacciners, traditional media is probably not the ideal outlet. Instead, personal contacts are an advantage here. Roccato & Russo (2021, p. 2) suggest health influencers, yet we would like to emphasize personal contact with experts, especially since it is easier to establish a dialogue instead of engaging in one-sided communication with influencers. We assume, that the public engagement of experts is very powerful and should be considered in future studies. If people meet an expert (such as scientists, doctors) in person who is willing to clarify or even correct false assumptions about vaccinations, this expert will stand out from the crowd of the ‘academic elite’ and might be more impactful in making a difference than traditional vaccination campaigns.

## Electronic Supplementary Material

Below is the link to the electronic supplementary material.

Supplementary Material 1

## Data Availability

The dataset for this study is available https://osf.io/f7urj/.

## Footnotes

1 In order to assure that these vaccines were discussed to varying intensity in the news, we conducted a news search using the LexisNexis database for journalistic news (https://advance.lexis.com). We used the German term for each vaccine without a time frame and limited the results to Austria and Germany. COVID-19, MMR, and seasonal influenza produced over 10.000 results, whereas the other vaccines had a considerably lower news coverage (TBE ~ 4.000; HPV ~ 2.600; MD ~ 1.300).

2 The results of the Model Fit, the Hosmer-Lemeshow-Test and the accuracy of the model fit are provided in table A1 in the supplement.
