# Supplementary material for: The Effect of Science-Related Populism on Vaccination Attitudes and Decisions
Source: J Behav Med. 2022 Jun 10;46(1-2):229–38. doi: 10.1007/s10865-022-00333-2 (PMC9185722; doi:10.1007/s10865-022-00333-2)
Supplement: Supplementary file 1 — Supplementary Material 1 [file 10865_2022_333_MOESM1_ESM.docx]

# Supplement

**Tab. A1 Additional tests for binomial logistic regression of vaccination status**

| **Vaccination** | **Model Fit** | **Hosmer-Lemeshow** | **Accuracy %** |
| --- | --- | --- | --- |
| Covid-19 | χ²(6) = 22.08, p<.001 | χ²(8) = 2.491, p = .962 | 64.8 |
| MMR | χ²(6) = 66.04, p<.001 | χ²(8) = 5.856, p = .663 | 85.1 |
| Seasonal influenza | χ²(6) = 5.036, p=.539 | χ²(8) =12.823, p = .118 | 76.8 |
| TBE | χ²(6) = 63.76, p<.001 | χ²(8) = 9.778, p = .281 | 67.5 |
| HPV | χ²(6) = 101.613, p<.001 | χ²(8) = 15.783, p = .046 | 81.3 |
| MD | χ²(6) = 121.191, p<.001 | χ²(8) = 7.780, p = .455 | 69.4 |
